# IrO*_x_*·*n*H_2_O with lattice water–assisted oxygen exchange for high-performance proton exchange membrane water electrolyzers

**DOI:** 10.1126/sciadv.adh1718

**Published:** 2023-06-23

**Authors:** Jun Xu, Huanyu Jin, Teng Lu, Junsheng Li, Yun Liu, Kenneth Davey, Yao Zheng, Shi-Zhang Qiao

**Affiliations:** ^1^ School of Chemical Engineering, The University of Adelaide, Adelaide, SA 5005, Australia.; ^2^ Institute for Sustainability, Energy and Resources, The University of Adelaide, Adelaide, SA 5005, Australia.; ^3^ Research School of Chemistry, The Australian National University, Canberra, ACT 2600, Australia.; ^4^ School of Chemistry, Chemical Engineering and Life Sciences, Wuhan University of Technology, Wuhan 430070, China.

## Abstract

The trade-off between activity and stability of oxygen evolution reaction (OER) catalysts in proton exchange membrane water electrolyzer (PEMWE) is challenging. Crystalline IrO_2_ displays good stability but exhibits poor activity; amorphous IrO*_x_* exhibits outstanding activity while sacrificing stability. Here, we combine the advantages of these two materials via a lattice water–incorporated iridium oxide (IrO*_x_*·*n*H_2_O) that has short-range ordered structure of hollandite-like framework. We confirm that IrO*_x_*·*n*H_2_O exhibits boosted activity and ultrahigh stability of >5700 hours (~8 months) with a record-high stability number of 1.9 × 10^7^ n_oxygen_ n_Ir_^−1^. We evidence that lattice water is active oxygen species in sustainable and rapid oxygen exchange. The lattice water–assisted modified OER mechanism contributes to improved activity and concurrent stability with no apparent structural degradation, which is different to the conventional adsorbate evolution mechanism and lattice oxygen mechanism. We demonstrate that a high-performance PEMWE with IrO*_x_*·*n*H_2_O as anode electrocatalyst delivers a cell voltage of 1.77 V at 1 A cm^−2^ for 600 hours (60°C).

## Teaser

Lattice-water-assisted oxygen exchange benefits IrOx·nH2O with excellent activity and stability for green hydrogen production.

## INTRODUCTION

Proton exchange membrane water electrolyzers (PEMWEs) are seen to be practically promising for hydrogen (H_2_) production with high current density (*1*, *2*). In PEMWE, the anodic oxygen evolution reaction (OER) is more inert together with a higher overpotential than the cathodic hydrogen evolution reaction. An anodic catalyst loading as high as 2 to 5 mg_Ir_ cm^−2^ to achieve high energy efficiency is usually required, which contradicts the low global production of Ir and limits PEMWE development (*2*–*4*). In addition, because of the harsh acidic and polarized environment, iridium oxides–based materials are unsatisfactory because of poor activity of crystalline rutile IrO_2_ or poor stability of amorphous IrO*_x_*. Typically, IrO_2_ with a high crystallinity exhibits poor activity in OER (*5*–*7*). For example, rutile IrO_2_ usually has a greater higher overpotential compared with other active OER catalysts such as RuO_2_, leading to a high cell voltage and high loading requirement (*5*). Amorphous IrO*_x_* exhibits increased active sites and activity while exhibiting inferior stability (*6*, *8*). A rational design for IrO_2_ catalysts with balance (trade-off) between activity and stability is therefore critical for continued development of practical low-Ir loaded PEMWEs (*9*, *10*).

The activity and stability of IrO_2_ are predicated on electrocatalytic mechanisms, e.g., adsorbate evolution mechanism (AEM) and lattice oxygen mechanism (LOM). Rutile IrO_2_ with high crystallinity has robust Ir─O bonds and therefore follows conventional AEM in which the oxygen molecule is generated from adsorbed water molecules (*11*, *12*). However, it has a theoretical overpotential of as high as 0.37 V, leading to high energy consumption (*13*). Substantial research is reported to boost activity; however, restriction of the inherent reaction mechanisms limits the improvement (*14*). Alternatively, activating lattice oxygen in IrO_2_ is feasible for boosting OER activity (*15*). For example, amorphous IrO*_x_* exhibits a substantially boosted catalytic activity in comparison with thermodynamically stable rutile IrO_2_ via triggering lattice-oxygen oxidation where unsaturated coordination oxygen atoms with weak Ir─O bonding are readily activated for OER (*6*, *16*). However, LOM initiates Ir─O bonding, leading to the accelerated degradation of electrocatalysts (*17*–*19*). As a result, conventional AEM limits activity, while LOM reduces stability of IrO_2_-based catalysts. It is important therefore to find a balance, or practical trade-off for behavior of oxygen exchange and to modify the reaction mechanism to involve more active lattice oxygen species in the reaction while maintaining a stable crystal structure (*8*).

Here, we demonstrate a lattice water–assisted short-range ordered iridium oxide** (IrO*_x_*·*n*H_2_O) as an OER electrocatalyst for highly stable acidic water oxidation. The developed IrO*_x_*·*n*H_2_O has a hollandite-like crystalline with abundant edge-sharing IrO_6_ octahedrons that accommodates structural water in its lattice, as lattice water, that contrasts with conventional crystalline or amorphous IrO_2_. We confirm via in situ characterizations that the lattice water is active oxygen species in sustainable and rapid oxygen exchange during OER and contributes to a modified lattice water–assisted pathway that is different to conventional AEM and LOM. As a result, we evidence that the IrO*_x_*·*n*H_2_O electrocatalyst exhibits notably boosted activity to rutile IrO_2_ and durability to amorphous IrO*_x_*. We confirm that the catalyst exhibits no apparent degradation following 5700 hours (~8 months) of test in a three-electrode cell in 0.1 M HClO_4_, and importantly, that it maintains excellent activity following 600 hours of pure water PEMWE operation at 60°C (at 1 A cm^−2^) with less Ir loading than a commercial membrane electrode assembly (MEA). We conclude that lattice water–assisted oxygen exchange is of practical benefit in design of anodic electrocatalysts for high-performance PEMWEs.

## RESULTS

### Structure analyses

**Fig. 1.**
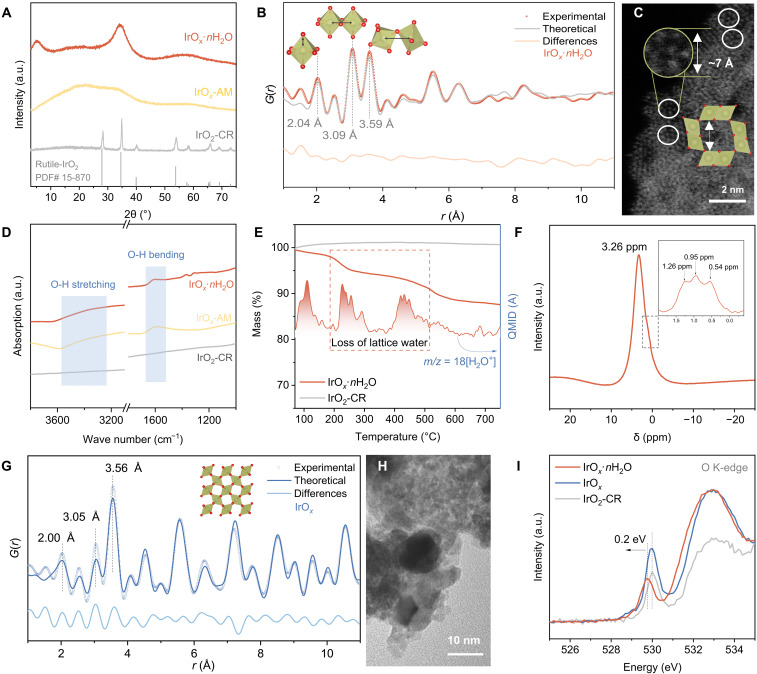
Structural analyses. (**A**) XRD patterns of IrO*_x_*·*n*H_2_O, IrO*_x_*-AM and IrO_2_-CR. a.u., arbitrary units. (**B**) PDF of IrO*_x_*·*n*H_2_O from PD data and theoretical calculations. (**C**) HAADF-STEM image of IrO*_x_*·*n*H_2_O with structure schematic for IrO*_x_*·*n*H_2_O inserted. (**D**) FTIR spectra of IrO*_x_*·*n*H_2_O, IrO*_x_*-AM and IrO_2_-CR. (**E**) TGA curves of IrO*_x_*·*n*H_2_O (with coupled MS signals) and IrO_2_-CR. QMID, quadrupole multiple ion detection. (**F**) ^1^H magic angle spinning ssNMR spectrum of IrO*_x_*·*n*H_2_O. (**G**) PDF of IrO*_x_*. (**H**) TEM image of IrO*_x_*. (**I**) Ex situ soft x-ray spectroscopic measurements at O K-edge of IrO*_x_*·*n*H_2_O, IrO*_x_* and IrO_2_-CR.

IrO*_x_*·*n*H_2_O was synthesized via a modified molten-salt method with rapid oxidation of IrCl_3_·*n*H_2_O in molten sodium nitrate under 360°C (*20*, *21*). A nanoparticle with a considerably smaller size than that for commercial rutile IrO_2_ (IrO_2_-CR, control sample) is obtained (fig. S1). An additional control sample, amorphous IrO*_x_* (IrO*_x_*-AM), that was synthesized did not exhibit regular morphology because of low crystallinity. The difference in crystallinity between the three IrO_2_-based samples is clear from x-ray diffraction (XRD) patterns. As is shown in Fig. 1A, IrO*_x_*·*n*H_2_O just shows three broad peaks. One peak ca. 5° confirms particle size of ca. 2 nm, which was consistent with the transmission electron microscopy (TEM) image (fig. S2). The other two broad peaks are apparent at ca. 34.4° and 59.0°, which is similar to amorphous IrO*_x_*-AM, while the positions are close to the main peaks of rutile IrO_2_-CR. These findings evidence that IrO*_x_*·*n*H_2_O with notably small particle size does not contain long-range ordered crystalline structure.

To determine the local structure, synchrotron-based powder diffraction (PD) with pair-distribution function (PDF) analysis was conducted (fig. S3) (*22*). As is seen in Fig. 1B, the PDF *G*(*r*) for IrO*_x_*·*n*H_2_O is different to that reported crystalline IrO_2_ (*23*), where clearly defined peaks can be found only at low *r* range, confirming short-range ordered characteristics. Although a low *r* range contains termination ripples arising from limited *Q*-range, the local structures can be analyzed from the three sharp pair correlation peaks <4 Å. The first peak ca. 2.04 Å is derived from the Ir-O pairs in IrO_6_ octahedra. The second peak at 3.09 Å evidences the edge-sharing Ir-Ir pairs within two neighbored IrO_6_ octahedra. The third peak, assigned to corner-sharing Ir-Ir pairs, positively shifts to 3.59 Å with increasing *r*. The intensity ratio for the edge- and corner-sharing Ir-Ir peaks for IrO*_x_*·*n*H_2_O confirms that the building blocks for IrO*_x_*·*n*H_2_O contain more edge-sharing IrO_6_ octahedra in contrast to the rutile structure that is dominated by the corner-sharing IrO_6_ octahedral (*23*). A hollandite-like structure (fig. S4) fits the IrO*_x_*·*n*H_2_O PDF data with good consistency within the 8-Å coherent domain size (Fig. 1B). The hollandite-like framework exhibits tunnels with a size of ca. 7 Å (*24*) that are confirmed in the high-angle annular dark-field scanning transmission electron microscopy (HAADF-STEM) image (Fig. 1C). The vibration of bonds between Ir and O was determined via Raman spectra (fig. S5). IrO*_x_*·*n*H_2_O exhibits similar vibrations to crystalline IrO_2_-CR, while the mode for B_2g_ is negatively shifted and the intensity of A_1g_ is decreased. These findings are attributed to its unique nanostructure nature with the stretched Ir─O bonds (*25*). IrO*_x_*-AM with the lowest crystallinity exhibited no apparent vibrations. These findings evidence that IrO*_x_*·*n*H_2_O is composed of short-range ordered hollandite-like clusters that are different to the conventional crystalline IrO_2_ and amorphous IrO*_x_*.

### Identification of structural lattice water molecules

The former structure analyses suggest a hollandite-like framework for IrO*_x_*·*n*H_2_O, which may host hydronium ions (defined as lattice water) in the tunnel to maintain thermodynamical stability of the whole framework (*23*). During the rapid molten salt synthesis, the concentration of water molecules in the muffle furnace increases as temperature rises. The absolute humidity of the muffle furnace at 360°C is greater than that at the initial temperature, 25°C (*20*), so the water molecules from the air can be readily incorporated into the reaction and enter the framework to balance the charge density and stabilize the structure. On the basis of this conjecture, the presence of lattice water was evidenced with findings from selected spectrometric technologies. Fourier transform infrared (FTIR) spectroscopy spectra show two characteristic peaks for water recorded for IrO*_x_*·*n*H_2_O (Fig. 1D), which are similar to IrO*_x_*-AM. One broad peak appears between 3600 and 3200 cm^−1^ and is attributed to the stretching of O-H and another peak at 1633 cm^−1^ is attributed to the bending of O-H. There are no signals of lattice water for IrO_2_-CR. Thermogravimetric analysis coupled with mass spectrometry (TGA-MS) confirms two mass loss stages for IrO*_x_*·*n*H_2_O. They are at 100° to 200°C and 200° to 500°C and are attributed to, respectively, the desorption of adsorbed water and loss of lattice water (Fig. 1E) (*26*). The ^1^H magic angle spinning solid-state nuclear magnetic resonance (ssNMR) spectrum for IrO*_x_*·*n*H_2_O confirmed existence of lattice water with a strong proton signal at 3.26 ppm (Fig. 1F). Note that it contains three tiny shoulder peaks (1.26, 0.95, and 0.54 ppm) that are likely contributed by different coordinated water in the lattice.

### Importance of lattice water for structural stability

To reveal the significance of lattice water for structural stability of the unique IrO*_x_*·*n*H_2_O framework, we annealed a fresh sample of IrO*_x_*·*n*H_2_O at 500°C for 2 hours in air to remove lattice water (fig. S6, resultant sample is IrO*_x_*). PD data and PDF analysis of IrO*_x_* demonstrate that the unique hollandite-like framework has collapsed and is converted to a general rutile phase, without the lattice water (Fig. 1G and fig. S7). The particle size of resultant IrO*_x_* without lattice water is quite larger than the pristine sample observed in the TEM images (Fig. 1H). The O K-edge characteristics from x-ray absorption near edge structure (XANES) for IrO*_x_*·*n*H_2_O exhibit one only pre-edge peak at 529.8 eV, that is negatively shifted by 0.2 eV from 530 eV for O^2−^. Following removal of the lattice water, the O K-edge of IrO*_x_* positively shifts back to 530 eV, which is a finding consistent with crystalline IrO_2_ (Fig. 1I). The coordination of lattice water likely leads to the slight negative shift of O K-edge in IrO*_x_*·*n*H_2_O (*26*, *27*), which is different from the amorphous IrO*_x_* with an extra signal at 529 eV attributed to active lattice oxygen (*28*). These findings together confirm the unique role of lattice water in stabilizing the unique short-range, ordered hollandite-like IrO*_x_*·*n*H_2_O.

### Electrocatalytic OER performance

**Fig. 2.**
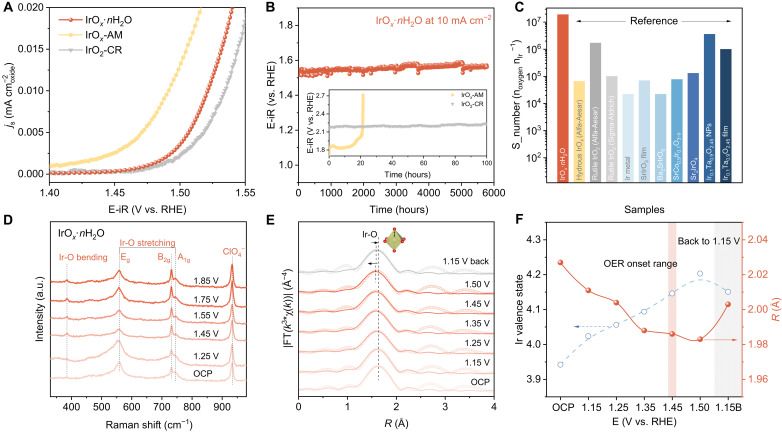
OER performance and structure characterization during OER. (**A**) ECSA normalized linear sweep voltammetry (LSV) curves. (**B**) Long-term stability of IrO*_x_*·*n*H_2_O, IrO*_x_*-AM, and IrO_2_-CR. (**C**) S_number calculated from (B) and content of dissolved Ir in electrolyte compared with reported Ir-based electrocatalysts. (**D**) Operando Raman spectra recorded in potential range of OCP ~1.85 V versus RHE for IrO*_x_*·*n*H_2_O. (**E**) *k*^3^-weighted operando FT-EXAFS profiles of IrO*_x_*·*n*H_2_O with no phase correction (circle, experimental data; line, fitted data). (**F**) Change in Ir valence state and Ir-O distance of IrO*_x_*·*n*H_2_O as a function of applied potential.

OER activity and stability for the three IrO_2_-based samples, with differing local structure, were assessed in a three-electrode system in O_2_-saturated 0.1 M HClO_4_. The amorphous IrO*_x_*-AM exhibits greatest electrochemical active surface area (ECSA) normalized specific activity (Fig. 2A and figs. S8 and S9). However, performance decays rapidly in <16 hours at 10 mA cm^−2^ in a chronopotentiometry test (Fig. 2B). The crystalline IrO_2_-CR exhibits good stability and poor activity. IrO*_x_*·*n*H_2_O exhibits greater specific activity than IrO_2_-CR and notably better stability than IrO*_x_*-AM, in which the overpotential at 10 mA cm^−2^ exhibits no apparent increase following 5700 hours (~8 months). In addition, the stability under large current (at 50 mA cm^−2^) has been further confirmed in fig. S10 that no apparent performance decreases after 200 hours. This is greater stability than even for most reported rutile IrO_2_-CR, and to our best knowledge, this is one of the most stable iridium oxides–based acidic OER electrocatalysts reported (table S1) (*6*, *8*, *29*–*38*). The difference in Tafel slope evidences that IrO*_x_*·*n*H_2_O has a different reaction mechanism to conventional amorphous IrO*_x_*-AM (e.g., LOM) and crystalline IrO_2_-CR (e.g., AEM) (fig. S11). In addition, IrO*_x_*·*n*H_2_O exhibits the greatest mass activity with more abundant active sites (fig. S12A). Activity for different loadings of IrO*_x_*·*n*H_2_O and IrO_2_-CR was determined in fig. S12B. No apparent change in performance is found at a tripled loading of IrO*_x_*·*n*H_2_O. This confirms that even with a low loading of IrO*_x_*·*n*H_2_O, a high activity is exhibited that is more efficient than crystalline IrO_2_-CR with a high loading. After removal of the lattice water, both the ECSA and intrinsic activity of each active site are decreased with the IrO*_x_* sample (fig. S13).

It is regarded generally that the consumption of unsaturated lattice oxygen during OER in amorphous IrO*_x_* results in fast dissolution of coordinated Ir under the polarization that leads to the poor stability (*8*, *19*, *39*). Therefore, the content of Ir dissolved from IrO*_x_*·*n*H_2_O in the anode electrolyte in H-cell was determined via inductively coupled plasma MS (ICP-MS). As is shown in fig. S14, a negligible dissolved Ir is observed in the electrolyte from the anodic cell up to 3000 hours. The stability number (S_number) of IrO*_x_*·*n*H_2_O is evaluated as a metric for electrocatalyst stability by calculating from the evolved oxygen gas per dissolved Ir atom in the electrolyte (*18*). As is seen in Fig. 2C, the S_number of IrO*_x_*·*n*H_2_O is as record high as 1.9 × 10^7^ n_oxygen_ n_Ir_^−1^, which is two orders of magnitude greater than that of commercial amorphous IrO*_x_* of (6.6 × 10^4^ n_oxygen_ n_Ir_^−1^) (*18*, *33*, *40*). This finding confirms that superior stability of the short-range ordered lattice water– incorporated IrO*_x_*·*n*H_2_O originates from a different mechanism from LOM that exhibits no dissolution of Ir compared with conventional amorphous IrO*_x_*.

In situ Raman spectra are presented as Fig. 2D and were determined to establish the structural change of IrO*_x_*·*n*H_2_O with increasing OER potential. As applied potential is increased to 1.85 V versus RHE (reversible hydrogen electrode), the bending and stretching modes of Ir─O bonds are maintained at the same position and no potential-dependent peaks appear, evidencing stable Ir-O structure of IrO*_x_*·*n*H_2_O. The post-test on the valence of O and Ir in IrO*_x_*·*n*H_2_O was conducted via, respectively, soft x-ray spectroscopy and x-ray absorption spectroscopy (XAS) (fig. S15). The O K-edge of IrO*_x_*·*n*H_2_O following OER is consistent with the fresh sample with a pre-edge peak at 529.8 eV, with no positive or negative shifts observed. This finding confirms the unique short-ranged framework accommodated with lattice water notably remains following OER. A meaningful “slight” decrease in intensity in the white-line peak for Ir L_3_-edge of IrO*_x_*·*n*H_2_O after OER was found that is likely the result of dynamical exchange of lattice water under potential. The hollandite-like structure after OER is apparent in the HAADF image (fig. S16). These findings confirm the stability of IrO*_x_*·*n*H_2_O in OER.

Operando XANES spectra were recorded under potential from open-circuit potential (OCP) to 1.50 V versus RHE to monitor structure evolution of IrO*_x_*·*n*H_2_O during OER in Fig. 2E (see setup in fig. S17). The valence change of Ir is determined via the intensity shift of the white-line peak (fig. S18), which increases with increased applied potential and recovers to a lower value following decrease of the bias to 1.15 V versus RHE (Fig. 2F), evidencing the stable structure of IrO*_x_*·*n*H_2_O coordinated with lattice water. The Ir─O bond change was assessed via fitting the Ir L_3_-edge Fourier transforms of extended x-ray absorption fine structure (FT-EXAFS) for IrO*_x_*·*n*H_2_O under differing potential (Fig. 2F, fig. S19, and table S2). The Ir─O bond “shrinks” from 2.027 to 1.983 Å when the potential increases from OCP to 1.50 V versus RHE. Following application of potential back to 1.15 V versus RHE, the bond changes to 2.003 Å; however, it does not recover to 2.011 Å (@1.15 V versus RHE). It is concluded therefore that the volume of the framework is slightly enlarged and stabilized because of shrinkage of I─O under electrochemical polarization that accommodates more water than the initial state. Operando XANES findings evidence the slightly decreased oxidation state of Ir following OER, which is likely the result of increased coordinated water from the dynamic and sustainable exchange from electrolyte under the bias.

### Role of lattice water in OER

**Fig. 3.**
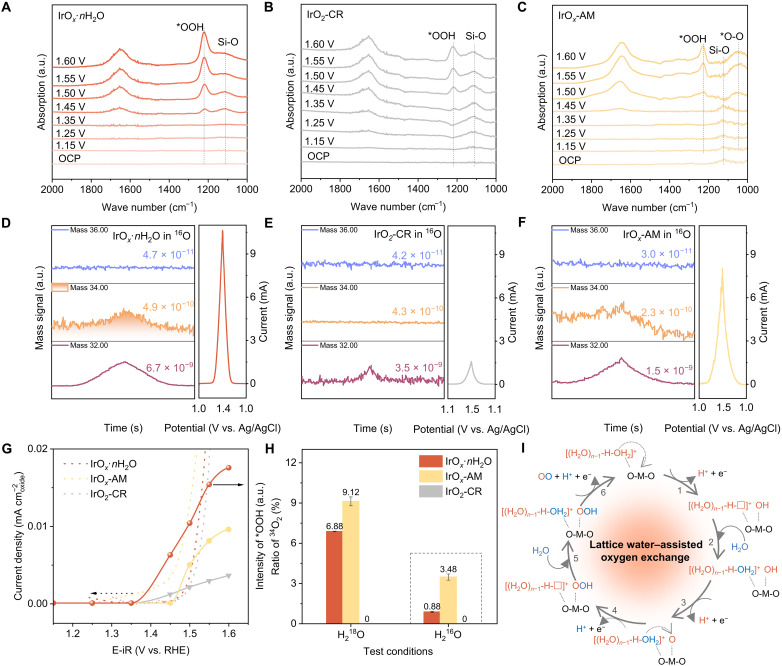
OER mechanism. In situ FTIR spectra in potential range of OCP ~1.6 V versus RHE of, (**A**) IrO*_x_*·*n*H_2_O, (**B**) IrO_2_-CR, and (**C**) IrO*_x_*-AM. Online DEMS signal of O_2_ products for IrO*_x_*·*n*H_2_O with corresponding CV curve for (**D**) IrO*_x_*·*n*H_2_O, (**E**) IrO_2_-CR, and (**F**) IrO*_x_*-AM. (**G**) Intensity of *OOH intermediate at different potentials. (**H**) Ratio of ^34^O_2_ in 0.05 M H_2_SO_4_ using H_2_^18^O or H_2_^16^O as solvents obtained on IrO*_x_*·*n*H_2_O, IrO*_x_*-AM, and IrO_2_-CR from DEMS results. (**I**) Schematic for oxygen evolution.

IrO*_x_*·*n*H_2_O with lattice water exhibits substantially boosted activity and stability. The role of lattice water in OER was established via in situ FTIR spectra (Fig. 3, A to C), and online differential electrochemical MS (DEMS) measurement (Fig. 3, D to F, and fig. S20). In the in situ FTIR spectra of IrO*_x_*·*n*H_2_O (Fig. 3A), in addition to two interfacial water peaks near 3460 and 1648 cm^−1^, an apparent signal peak near wave number 1222 cm^−1^ is observed under a potential of 1.45 V (versus RHE). It increases with potential that is attributed to generation of *OOH (*41*). This trend is similar to that for behavior of rutile IrO_2_-CR (Fig. 3B) that follows conventional AEM. For amorphous IrO*_x_*-AM (Fig. 3C), there are no signals of *OOH at 1.45 V, even though the current surpasses that of the IrO_2_-CR (Fig. 3G). As potential increases, a broad peak appeared at 1000 to 1100 cm^−1^ that is attributed to the signal of *OO (*42*), evidencing dominance of LOM in IrO*_x_*-AM under low overpotential. *OOH is observed when the potential is 1.50 V because high potential promotes AEM. Notably, the appearance of *OOH intermediates without *OO on IrO*_x_*·*n*H_2_O evidences that it follows a mechanism similar to AEM in which oxygen is generated from one water molecule dissociating and *O combining with another dissociated water molecule to form *OOH, and oxygen is released (*43*). As is seen in Fig. 3G, the intensity of *OOH recorded on IrO*_x_*·*n*H_2_O is considerably stronger than that recorded for IrO_2_-CR following conventional AEM and IrO*_x_*-AM dominated by conventional LOM at potential of 1.45 V, evidencing faster oxygen exchange on IrO*_x_*·*n*H_2_O.

Online DEMS combining isotope labeling was applied to establish the role of lattice water in oxygen exchange and formation of oxygen molecules. Oxygen products including ^32^O_2_(^16^O^16^O), ^34^O_2_(^16^O^18^O), and ^36^O_2_(^18^O^18^O) were collected and analyzed to confirm the source of oxygen in generated oxygen molecules, either from external adsorbed water or from the lattice in the oxides. These experiments were carried out in two steps, namely, step 1 is to make ^18^O labeling on the oxygen of IrO*_x_*·*n*H_2_O, IrO*_x_*-AM, and IrO_2_-CR in both lattice oxygen and lattice water by cyclic voltammetry (CV) scanning in the electrolyte with H_2_^18^O as solvent (fig. S20). In this step, ^36^O_2_ is the main component of anodic gas products together with some ^34^O_2_ derived from the impurity ^16^O of H_2_^18^O and the oxygen from the oxides. As presented in Fig. 3H, ^34^O_2_ obtained with IrO*_x_*·*n*H_2_O as OER electrocatalyst shows a ratio of 6.88%, while that for IrO*_x_*-AM is 9.12% and for IrO_2_-CR exhibits zero ^34^O_2_. These findings evidence that different oxygen from oxides of IrO*_x_*·*n*H_2_O and conventional amorphous IrO*_x_*-AM are involved in the oxygen exchange.

Step 2 is to determine how much labeled oxygen from the catalysts participates in OER under 0.05 M H_2_SO_4_ (H_2_^16^O as solvent) with the labeled IrO*_x_*·*n*H_2_O and control samples from step 1. As can be seen (Fig. 3, D to F and H), 0.88% of ^34^O_2_ is obtained with IrO*_x_*·*n*H_2_O, which is substantially less than that for IrO*_x_*-AM of 3.48% and more than that for rutile IrO_2_-CR of 0%. The higher ratio of ^34^O_2_ from amorphous IrO*_x_*-AM is attributed to the participation of lattice oxygen in OER as LOM occurs. For the rutile IrO_2_-CR, with only AEM happens, adsorbed water only is involved in oxygen evolution, and no labeled oxygen in oxides is available to support oxygen exchange. The IrO*_x_* sample after removing lattice water confirmed that rutile iridium oxide without lattice water incorporated follows a conventional AEM mechanism, in which no lattice oxygen is involved in oxygen evolution, excluding a natural ^34^O_2_ ratio of 0.21% because of 0.2% natural abundance of H_2_^18^O (*44*) (fig. S21). For IrO*_x_*·*n*H_2_O, as is confirmed in the in situ IR spectra, the presence of *OOH evidences AEM-like route and rules out conventional LOM, and a higher ^34^O_2_ derived from the labeled oxygen of IrO*_x_*·*n*H_2_O is specially assigned the ^18^O source to the labeled lattice water.

On the basis of these findings from in situ FTIR and online DEMS, we hypothesize a modified mechanism with lattice water–assisted oxygen exchange for IrO*_x_*·*n*H_2_O in acid OER (Fig. 3I) that is different to conventional AEM and LOM. Namely, initially lattice water is accommodated in the framework of IrO*_x_*·*n*H_2_O in the form of hydronium ions, which are readily nucleophilic attacked by adsorbed water under anodic potential (*23*). When the OER initiates, the lattice water molecules close to the active site (Ir), marked in orange-color in Fig. 3I, can easily move to the metal site and be dissociated to form *OH intermediates to participate in following oxygen exchange. This leaves a vacant position for the subsequent nucleophilic attack of water from electrolyte (step 1). The external adsorbed water rapidly fills the vacant position of dissociated lattice water to stabilize the structure (step 2), and the newly filled water is dissociated rapidly to generate oxygen molecules on Ir as illustrated in steps 3 to 6, which are similar to AEM route. Notably, step 1 is similar to that in LOM, in that the lattice water directly participates in oxygen exchange, thereby boosting the intrinsic activity of IrO*_x_*·*n*H_2_O. However, the lattice water is sustainably supplied so that the structure of IrO*_x_*·*n*H_2_O maintains excellent durability, in contrast to conventional LOM that occurs with unrecoverable destruction on catalysts. Therefore, a faster and more sustainable oxygen exchange than with conventional AEM and LOM occurs with support of lattice water to deliver measurable boosted activity and stability.

### PEMWE performance

**Fig. 4.**
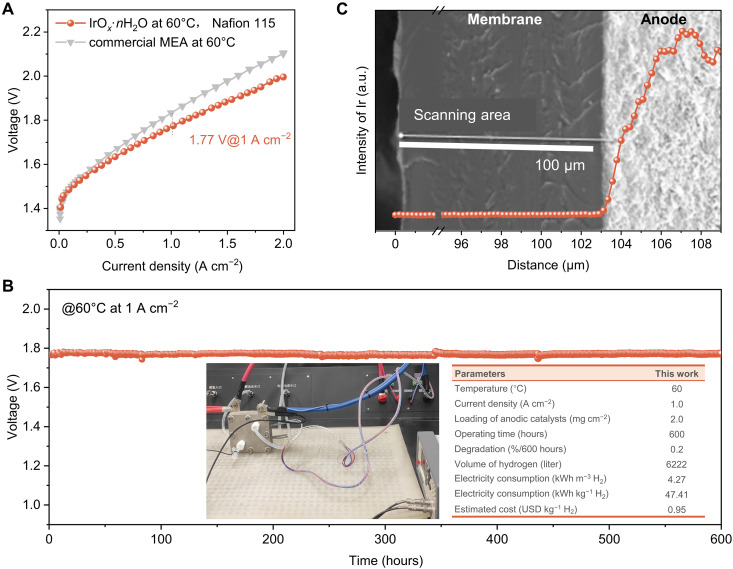
PEM performance. (**A**) Steady-state polarization curves of the PEM electrolyzer using IrO*_x_*·*n*H_2_O and commercial IrO_2_ as anodic catalysts coated on Nafion 115 membrane operated at 60°C. (**B**) Chronopotentiometric curve of the PEM electrolyzer using IrO*_x_*·*n*H_2_O with digital photographs of PEMWE device and parameters. (**C**) SEM image of cross section of MEA and signals for Ir near the anode catalyst/membrane interface via EDS linear scanning.

Performance of IrO*_x_*·*n*H_2_O was demonstrated in a commercial PEM electrolyzer at 60°C for deionized (DI) water splitting. The electrolyzer is shown as fig. S22. The MEA was customized with an anodic IrO*_x_*·*n*H_2_O loading of 2 mg cm^−2^ on Nafion 115 and 3 mg cm^−2^ of platinum-black coated on the cathode. A commercial MEA was prepared for direct comparison with 3 mg cm^−2^ of IrO_2_ in the anode. With less loading of IrO*_x_*·*n*H_2_O working as anodic catalysts, the customized MEA exhibits a cell voltage of 1.77 V at 1 A cm^−2^, which outperforms the commercial MEA by 60 mV (Fig. 4A). IrO*_x_*·*n*H_2_O exhibits highly substantial stability in PEM applications, with no apparent increase (ca. 0.2%/600 hours) in cell voltage at 1 A cm^−2^ following 600 hours to produce 0.56 kg [6222 liters at standard temperature and pressure (STP)] of H_2_ as is seen from Fig. 4B. The energy consumption is just 4.27 kWh m^−3^ H_2_ (equated to 47.41 kWh kg^−1^ H_2_) at 1.0 A cm^−2^, which is less than that for reported commercial PEM electrolyzers of 4.5 to 5.0 kWh m^−3^ H_2_ (50 to 55.56 kWh kg^−1^ H_2_) (*45*). The estimated cost of this PEMWE is USD 0.95 per kg H_2_ (*37*), which is close to the 2030 goal of USD 1.0 per kg H_2_ of the US Hydrogen Earthshot initiative (*46*). A comparative summary of PEMWE performance between IrO*_x_*·*n*H_2_O with selected typical anodic electrocatalysts is presented as table S3 (*37*, *38*, *47*–*51*). These findings combined confirm the short-range ordered IrO*_x_*·*n*H_2_O with modified oxygen exchange assisted by lattice water is practical for PEMWE.

Following operation at 1 A cm^−2^ for 600 hours, the post-test for IrO*_x_*·*n*H_2_O in MEA was determined via Raman spectroscopy (fig. S23). A similar Raman spectrum for IrO*_x_*·*n*H_2_O was collected that exhibited the same vibrations of Ir─O bonds as prior to the PEMWE test. This finding evidences that there is no apparent structural change following PEMWE operation. As the dispersed nanoparticles and a diffuse layer of Ir after prolonged operation of the PEMWE at high current densities have been previously reported in the catalyst/membrane interface (*52*), the dissolution and precipitation of Ir species were thus determined via a scanning electron microscope (SEM) and energy-dispersive spectrometer (EDS) (Fig. 4C). No dispersed Ir nanoparticles signals were detected in the SEM cross section. This finding confirms a negligible dissolution of Ir following operation of PEMWE at 1 A cm^−2^ for 600 hours. It is concluded therefore the short-range ordered IrO*_x_*·*n*H_2_O, with a modified mechanism assisted by lattice water, is stable in PEMWE under high current density, making it attractive for application in practical devices.

## DISCUSSION

In summary, we demonstrate a short-range ordered IrO*_x_*·*n*H_2_O that combines advantage of crystalline and amorphous IrO_2_ for an active and durable OER. PDF, together with in situ spectroscopic analyses, evidence that the IrO*_x_*·*n*H_2_O has a hollandite-like framework that accommodates abundant lattice water. The lattice water sustainably participates in oxygen exchange so that OER activity is highly notably boosted without apparent attenuation following 5700 hours (~8 months) under polarization. With the modified oxygen exchange assisted by lattice water, IrO*_x_*·*n*H_2_O maintains stability of crystalline IrO_2_ and exhibits concurrently excellent activity of amorphous iridium oxide. In a PEMWE application, IrO*_x_*·*n*H_2_O as anodic catalyst delivers a cell voltage of 1.77 V @ 1 A cm^−2^ maintained for 600 hours. This is with less Ir-loading than commercial MEA and an energy consumption of just 4.27 kWh m^−3^ H_2_ at 1.0 A cm^−2^, together with an estimated cost of USD 0.95 per kg H_2_. Lattice water–assisted oxygen exchange is therefore of practical benefit in the design of anodic electrocatalysts for high-performance PEMWE. Findings will be of interest to researchers and manufacturers in design of equipment and applications.

## MATERIALS AND METHODS

### Chemicals

Iridium(III) chloride hydrate (IrCl_3_·*n*H_2_O, reagent grade), sodium nitrate (NaNO_3_, ReagentPlus, ≥99.0%), lithium hydroxide (LiOH, reagent grade, 98%), and iridium(IV) oxide (IrO_2_-CR, 99.9%) were purchased from Sigma-Aldrich. DI water was supplied by the Milli-Q Benchtop laboratory water purification systems (Sigma-Aldrich).

### Material synthesis

IrO*_x_*·*n*H_2_O was synthesized via a modified molten-salt method (*20*, *21*). Five grams of NaNO_3_ was melted at 360°C in a muffle furnace, to which 40 mg of IrCl_3_·*n*H_2_O was added rapidly with continued reaction for 5 min. The crucible was removed and cooled naturally to room temperature (RT). Salts were removed by DI water, and IrO*_x_*·*n*H_2_O was separated via filtration and dried at 60°C overnight. IrO*_x_* was obtained by annealing IrO*_x_*·*n*H_2_O in air at 500°C for 2 hours, and the lattice water was completely removed.

Amorphous IrO*_x_*-AM was synthesized using reported methods (*53*). IrCl_3_·*n*H_2_O (120 mg) and 77 mg of LiOH were added to 4 ml of DI water and stirred overnight to generate a blue-color solution. An additional 4 ml of DI water was added, and the mix was heated to reflux for 3 hours. Last, the product was washed with hot DI water thoroughly and dried naturally.

### Characterization

XRD data were recorded on a Rigaku MiniFlex 600 XRD using Cu Kα (1.54 Å, 40 kV, 15 mA) x-ray source. PD patterns were collected from the Powder Diffraction beamline at the Australian Synchrotron with the wavelength (λ) of 0.5903 Å. The PDFgetX3 was used to convert the x-ray PD data to the atomic PDF (*54*). Experimental PDFs were analyzed using the software package, PDFgui (*55*). O K-edge spectra were collected on the Soft X-ray Spectroscopy beamline at the Australian Synchrotron, and all data were calibrated with reference to the standard foil. HAADF-STEM images were recorded on the aberration-corrected FEI Titan Themis operating at 200 kV. SEM images were collected using a FEI QUANTA 450 FEG Environmental SEM OPERATING at 10 kV. TEM images were recorded on Phillips CM200 operating at 200 kV. TGA-MS analyses were carried out with a NETZSCH STA 449 F5 Jupiter. TGA-MS was operated via heating ca. 5 mg of sample under high-purity nitrogen with 10 ml min^−1^, from RT to 750°C at a rate of 5 K min^−1^ (for improved signal). ^1^H ssNMR spectrum was determined at 700 MHz with a spinning rate of 40 kHz at RT using a Phoenix NMR triple resonance broadband probe. FTIR spectrum for powder (pretreated at 120°C overnight) was collected on a Nicolet 6700 Fourier Transform Infrared Spectrometer. Ex situ Raman spectra were recorded on Renishaw Raman spectroscopy with a 50× objective and a laser wavelength of 532 nm.

### Electrochemical characterization

Electrochemical measurements were carried out in a three-electrode system in O_2_-saturated 0.1 M HClO_4_ with a CHI-760E electrochemical workstation under RT. Four milligrams of catalyst was dispersed in a 1 ml of mixture of water and ethanol (AR) (*V*_water_/*V*_ethanol_ = 4/1), 40 μl of 5 wt % Nafion solution was added and the mix ultrasonicated for 60 min to obtain a homogeneous ink. The ink (7.6 μl) was dropped on a rotating disk electrode (Pine Research Instrument) with a diameter of 5 mm and dried naturally for the working electrode. For the stability test on fluorine-doped tin oxide (FTO) and platinized titanium felt (for large density test), ca. 200 μl of catalyst was sprayed on an area of 0.5 cm^2^, and loading of 1.5 mg cm^−2^ was controlled. A Pt-wire was selected as the counter and Ag/AgCl as the reference electrode. The working electrode was cyclically scanned from 1.0 to 1.55 V (versus RHE) to reach a stable state for pre-activation for OER activity determination. Linear sweep voltammetry curves were collected with a scanning rate of 2 mV s^−1^. Potentials were corrected by 100% *i*R compensation. Electrochemical active surface area was estimated from the electrochemical double-layer capacitance (C_dl_) from Eq. 1 (*56*). The value for C_dl_ was determined via CV scanning in a non-Faradic region from 1.0 to 1.1 V versus Ag/AgCl with scanning rate, 20, 40, 60, 80, and 100 mV s^−1^. The current at 1.05 V versus Ag/AgCl exhibited a linear relationship with scanning rate. The value of the slope was determined from fitting of the data to obtain C_dl_. The specific current density per ECSA (*j*_s_) was computed via normalizing the current by ECSA, namely

$$\mathrm{ECSA}=\frac{C_{\mathrm{dl}}}{0.035\;\mathrm{mF}\;{\mathrm{cm}}^{-2}}$$ (1)

Mass activity (*j_m_*) was determined from Eq. 2, where *j_m_* is the current density normalized by the geometric area, *A*_geo_ is the electrode area and *m*_Ir_ is the iridium loading mass of electrocatalyst on electrode.

$$j_{m}=\frac{j_{\mathrm{geo}}\times A_{\mathrm{geo}}}{m_{\mathrm{Ir}}}$$ (2)

The stability test of the catalyst was determined at a current density of 10 mA cm^−2^ at RT. A stirring bar was used in the electrolyte to remove bubbles from the surface of the electrode and for fast proton transfer during the test. *E*-t curves were 100% *i*R-compensated. Commercial crystalline and amorphous IrO_2_ were similarly prepared for stability comparison.

Potentials were calibrated to an RHE in H_2_-saturated 0.1 M HClO_4_ (*57*).

$$E_{\mathrm{RHE}}=E_{\mathrm{Ag}/\mathrm{AgCl}}+0.25\;V$$ (3)

The stability number (S_number) was computed from Eq. 4 (*18*)

$$S\_\mathrm{number}=\frac{n_{\mathrm{oxygen}}}{n_{\mathrm{iridium}}}$$ (4)

where *n*_oxygen_ is the molar number of oxygen produced during a time and *n*_iridium_ is the molar number of dissolved Ir in electrolyte determined via ICP-MS.

### ICP-MS analyses of Ir dissolution

The dissolution of Ir during OER was quantified via ICP-MS (Agilent 8900 ICP-QQQ). An H-cell was applied to separate the working and counter electrode. The working electrode was prepared by spraying the catalyst on FTO with a loading of 1.5 mg cm^−2^. One hundred milliliters of 0.1 M HClO_4_ was used as electrolyte. Two milliliters of the electrolyte from the anodic cell following electrolysis (@ 10 mA cm^−2^) at 0, 0.5, 1, 2, 4, 24, 48, 100, 200 and 3000 hours was collected for ICP-MS measurement.

### Online DEMS measurement

DEMS (The Hidden HPR-40 DEMS) was used to determine the OER mechanism combined with ^18^O isotope labeling. A customized flow cell was used and the electrolyte pumped through the cell at 50 ml min^−1^. The working electrode was prepared by coating Au onto a polytetrafluoroethylene (PTFE) film. A loading of 0.2 mg cm^−2^ catalyst was dropped on the Au-film and dried at 60°C. A saturated Ag/AgCl was the reference and Pt-wire the counter electrode. For greater purity of H_2_^18^O, we selected 98% H_2_SO_4_ and diluted it to pH = 1 as the electrolyte for the DEMS isotope labeling. CV scanning in the range 1.0 to 1.4 V (IrO*_x_*·*n*H_2_O) /1.50 V(IrO*_x_*-AM, IrO_2_-CR) (versus Ag/AgCl) for 5 cycles at a scanning rate of 5 mV s^−1^ was applied under H_2_^18^O supporting 0.05 M H_2_SO_4_. The working electrode and the whole cell were washed with DI water thoroughly to remove ^18^O and then dried at 60°C. ^18^O-labeled electrodes were prepared and CV cycles performed in 0.05 M H_2_SO_4_ (H_2_^16^O as solvent) with the same potential window and scan rate. During OER, oxygen with different molecular masses was collected and determined via mass spectroscopy. With the signals of ^34^O_2_, the behavior of oxygen exchange during OER can be established. Experiments were repeated three times.

### In situ FTIR

In situ FTIR was carried out on Nicolet iS50 FTIR Spectrometer to determine the potential-dependent intermediates and reaction mechanism. A PIKE jackfish cell was used and O_2_-saturated 0.1 M HClO_4_ was selected as electrolyte. A catalyst loading of 0.2 mg cm^−2^ was dropped on an Au-coated Si ATR wafer, which dried naturally and acted as a working electrode. A saturated Ag/AgCl was the reference and a Pt-wire counter electrode. The background was subtracted under open circuit potential. Spectra between 1000 and 4000 cm^−1^ were collected at differing potential with polarization in advance for at least 5 min to reach a stable current.

### In situ Raman spectroscopy

In situ Raman spectroscopy was conducted on Renishaw Raman spectroscopy with a special 60× objective that was immersed in the electrolyte and a laser wavelength of 532 nm. Collection was at 10 s of exposure with 10 exposures. A screen-printed chip electrode from Pine Research Instrumentation was used. Prepared ink (10 μl) was dropped on the chip and dried at RT, and 0.1 M HClO_4_ was applied as electrolyte. Spectra were collected following removal of bubbles at different potential with polarization in advance for 10 min. Measurement at each potential was carried out at three differing points.

### XAS measurement

XAS data were determined with the XAS beamline at the Australian Synchrotron ANSTO. Ex situ XAS measurements were determined via transmission mode in the RT chamber. Operando XAS measurements were determined in three repeats to confirm findings. A homemade operando cell and CHI-760E electrochemical workstation were used. IrO*_x_*·*n*H_2_O loaded on carbon paper was used as the working electrode. Ag/AgCl was used as the reference and Pt-wire as counter electrode. Oxygen-saturated 0.1 M HClO_4_ electrolyte was pumped through the cell at 50 ml min^−1^ to remove any in situ generated bubbles. Operando Ir-L_3_ XANES and FT-EXAFS spectra were recorded in fluorescence mode at RT. Data were analyzed and processed via Athena and Artemis software following standard procedure using the Demeter program package (0.9.26) (*58*).

### PEM measurement

The PEMWE measurements were conducted via the test system, JNR (Wuhan Jingneng Electronic Technology Co., Ltd.). Catalysts were prepared as a MEA with an anodic loading of 2 mg cm^−2^ (IrO*_x_*·*n*H_2_O) on Nafion 115 and 3 mg cm^−2^ of Pt black coated on the cathode. A commercial MEA was purchased from Fuel Cell Store for comparison with 3 mg cm^−2^ of IrO_2_ coated on the anode and 3 mg cm^−2^ of Pt black coated on the cathode. The cell was integrated via pressing two Ti bipolar plates, diffusion layers, and MEA. A Ti-fiber felt (0.25-mm thickness, 78% porosity, Bekaert) was used as the anodic gas diffusion layer (GDL), and carbon paper (TGP-H-060, Toray Industries, Inc) as the cathodic GDL. The active area of MEA was controlled at 25 cm^2^ (5 cm*5 cm). For the PEM test, the temperature was controlled at 60°C, and preheated DI water (18.2 Mohm cm, 25°C) was kept flowing through the cell at 50 ml min^−1^. The water was added to fill a 10-liter tank of the test system and was supplemented per 5 days. Activation was carried out prior to the test with a prepolarization at 1 A cm^−2^ for 1 hour. The steady-state polarization curve was determined via the galvanostatic method, and each point was recorded until the cell voltage was stable. The stability test in PEM ran at 1 A cm^−2^ for 600 hours.

### Determination of dissolved and precipitated Ir in MEA

The post-test of MEA for determination of dissolution and precipitation of Ir was conducted using SEM-EDS. The MEA cut-outs were immersed in liquid nitrogen for 1 min to obtain brittle fracture. The fracture was prepared for SEM imaging by carbon coating. Elemental mapping by EDS with linear scanning at an acceleration voltage of 20 kV was used to record dissolved Ir species in the membrane.

### Estimation of hydrogen produced and cost in PEMWE 

The calculation of hydrogen cost in PEMWE was based on (*37*). Specifically,

$$\text{Mass of}\;H_{2}=\frac{j(\text{current density})\times A(\text{electrolyzer area})\times t\;(\text{working time})\times \text{Molar mass}\;H_{2}}{2\times F}=\frac{1\;A\;{\mathrm{cm}}^{-2}\times 25\;{\mathrm{cm}}^{2}\times 600\;\mathrm{hours}\times 3600\frac{\mathrm{hours}}{s}\times 2\;g\;{\mathrm{mol}}^{-1}}{2\times 96485\;c\;{\mathrm{mol}}^{-1}}=560\;g$$ (5)

$$\text{Volume of}\;H_{2}=\frac{m}{\rho }=\frac{560\;g\;}{0.09\;g\;{\mathrm{liter}}^{-1}}=6222\;\mathrm{liters}$$ (6)

$$\text{Energy consumption}=\frac{1.77\;V\times 25\;A\times 600\;\mathrm{hours}}{0.56\;\mathrm{kg}\;H_{2}}=\frac{47.41\;\mathrm{kWh}}{\mathrm{kg}\;H_{2}}=\frac{4.27\;\mathrm{kWh}}{m^{3}\;H_{2}}$$ (7)

$$\text{Cost per kilogram of}\;H_{2}=\text{energy consumption}\times \text{electricity bill}=\frac{1.77\;V\times 25\;A\times 600\;\mathrm{hours}}{0.56\;\mathrm{kg}\;H_{2}}\times \frac{\mathrm{USD}\;0.02}{\mathrm{kWh}}=\frac{\mathrm{USD}\;0.95}{\mathrm{kg}\;H_{2}}$$ (8)
