# Supplementary material for: IrOx·nH2O with lattice water–assisted oxygen exchange for high-performance proton exchange membrane water electrolyzers
Source: Sci Adv. 2023 Jun 23;9(25):eadh1718. doi: 10.1126/sciadv.adh1718 (PMC10289644; doi:10.1126/sciadv.adh1718)
Supplement: Supplementary file 1 — Figs. S1 to S23 Tables S1 to S3 [file sciadv.adh1718_sm.pdf]

Science Advances
AAAS

Supplementary Materials for

$IrO_x \cdot nH_2O$ with lattice water–assisted oxygen exchange for high-performance proton exchange membrane water electrolyzers

Jun Xu *et al.*

Corresponding author: Yao Zheng, yao.zheng01@adelaide.edu.au; Shi-Zhang Qiao, s.qiao@adelaide.edu.au

Sci. Adv. **9**, eadh1718 (2023)
DOI: 10.1126/sciadv.adh1718

This PDF file includes:

Figs. S1 to S23
Tables S1 to S3

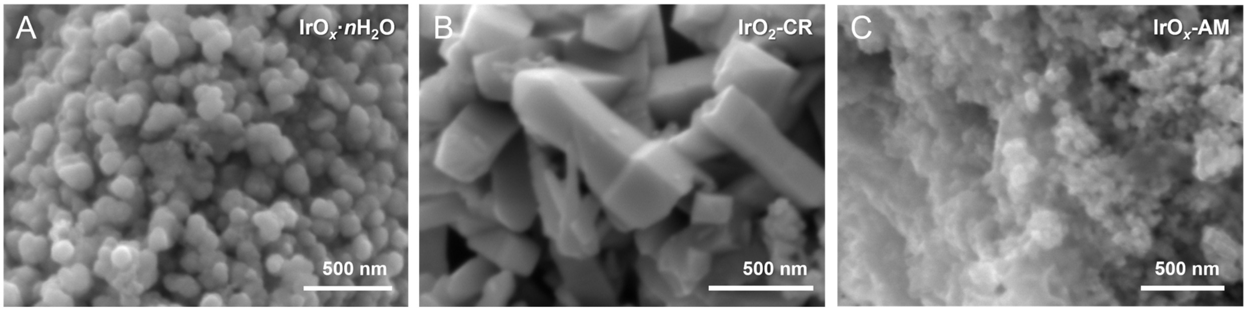

Fig. S1. SEM images. (**A**) $IrO_x \cdot nH_2O$, (**B**) IrO_2-CR and (**C**) IrO_x-AM.

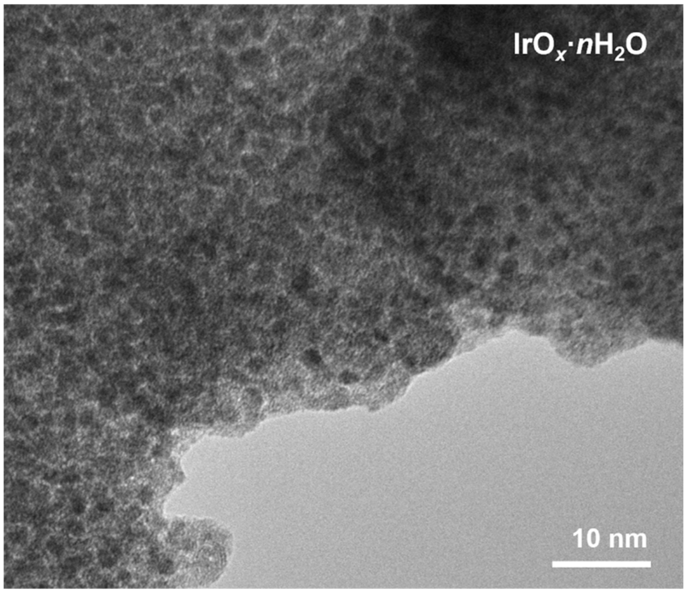

Fig. S2. TEM image of $IrO_x \cdot nH_2O$.

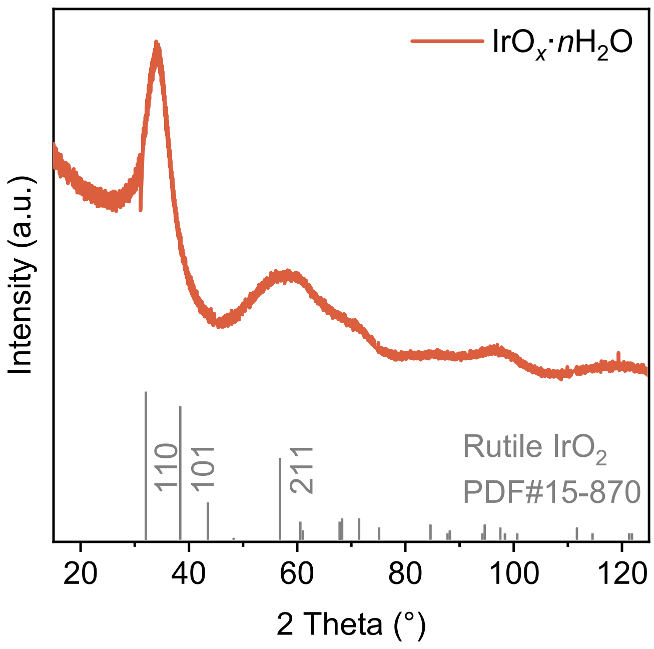

Fig. S3. Powder diffraction pattern of $IrO_x \cdot nH_2O$. All PD data in this work were converted to the range of Cu Kα (1.54 Å) corresponding to Bragg's Law.

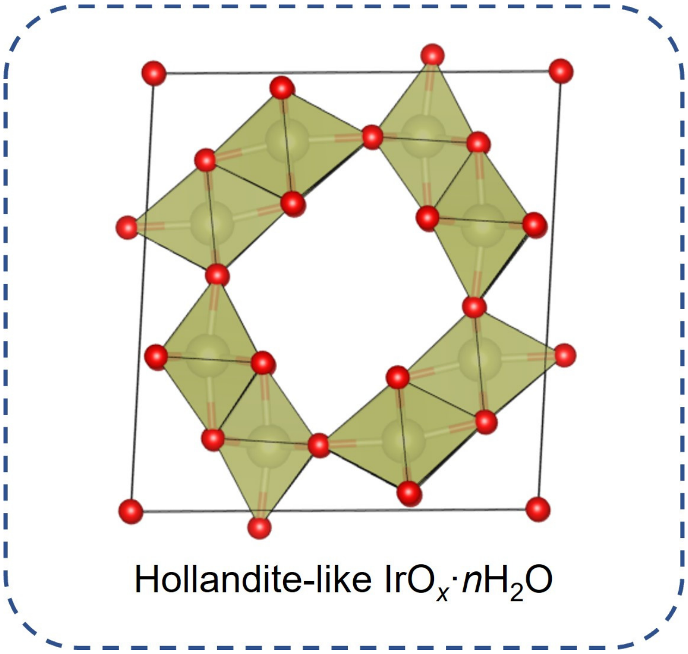

Fig. S4. Schematic for structure for hollandite-like $IrO_x \cdot nH_2O$.

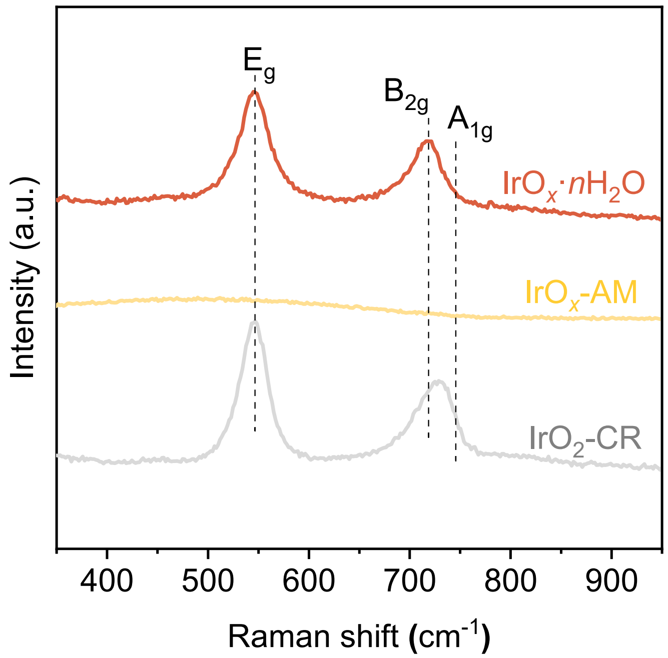

Fig. S5. Raman spectra of $IrO_x \cdot nH_2O$, IrO_2-AM and IrO_x-CR.

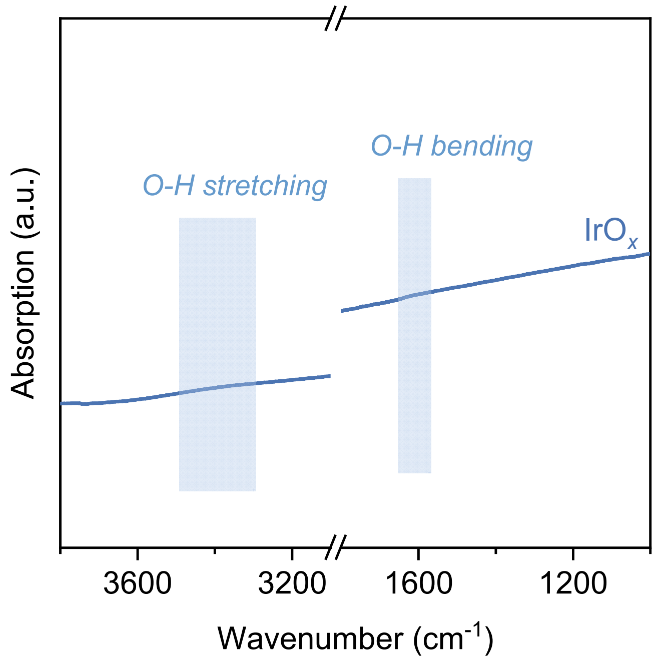

Fig. S6. FTIR spectrum of IrO_x.

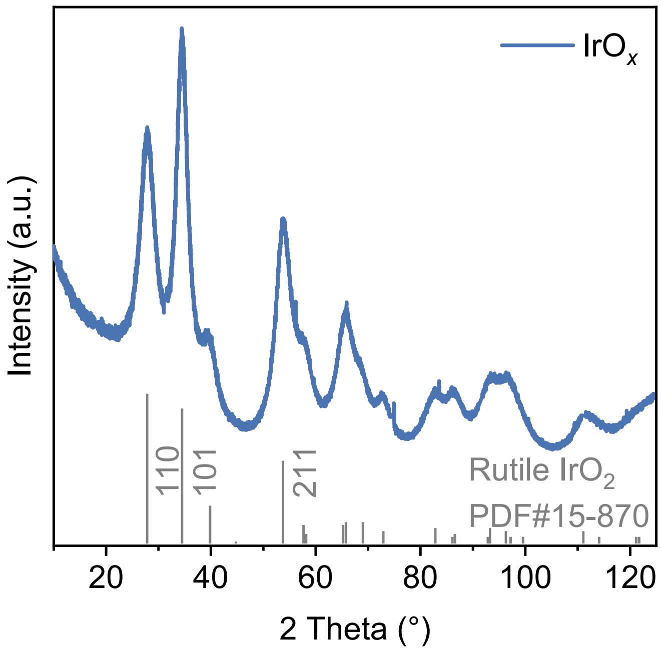

Fig. S7. Powder diffraction pattern of IrO_x.

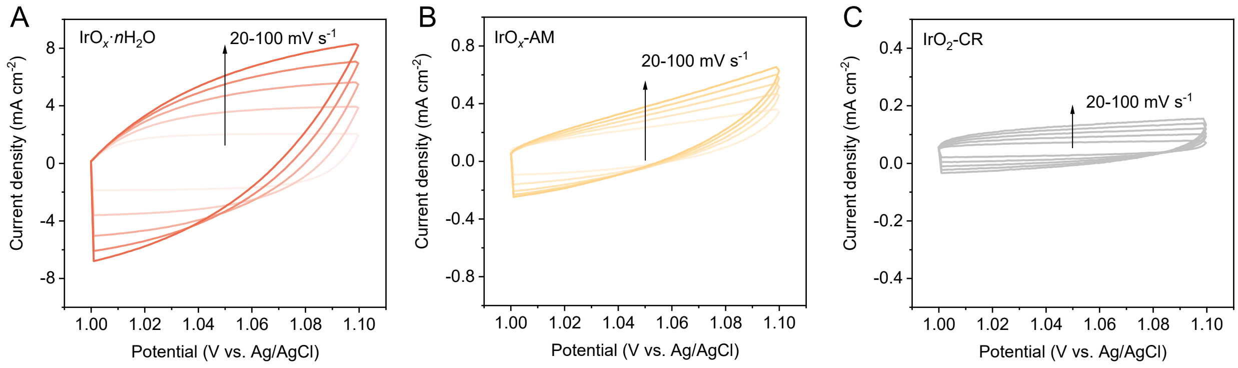

Fig. S8. CV curves at different scan rates in a non-Faradic region. (**A**) $IrO_x \cdot nH_2O$, (**B**) IrO_x-AM and (**C**) IrO_2-CR.

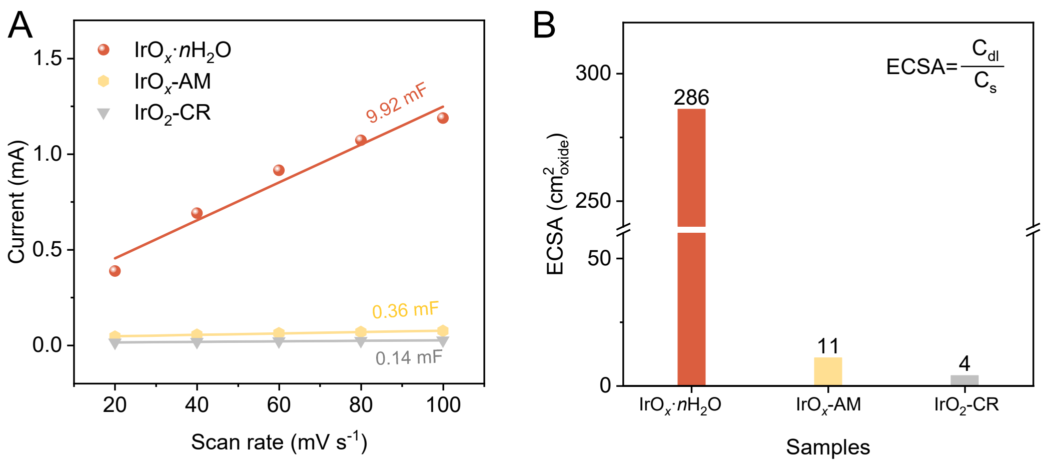

Fig. S9. Electrochemical active area (ECSA) analyses. (**A**) Double-layer capacitance (C_{dl}) represented by curve slope and (**B**) ECSA comparison.

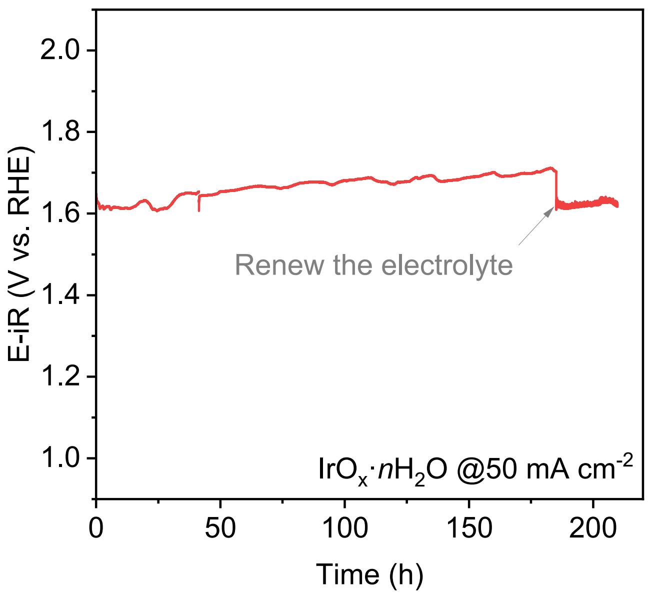

Fig. S10. Chronopotentiometry curve of $IrO_x{\cdot}nH_2O$ at 50 mA cm^{-2} in the three-electrode system. A platinized titanium felt as the stable substrate of the working electrode was used to increase the contact between catalysts and substrates.

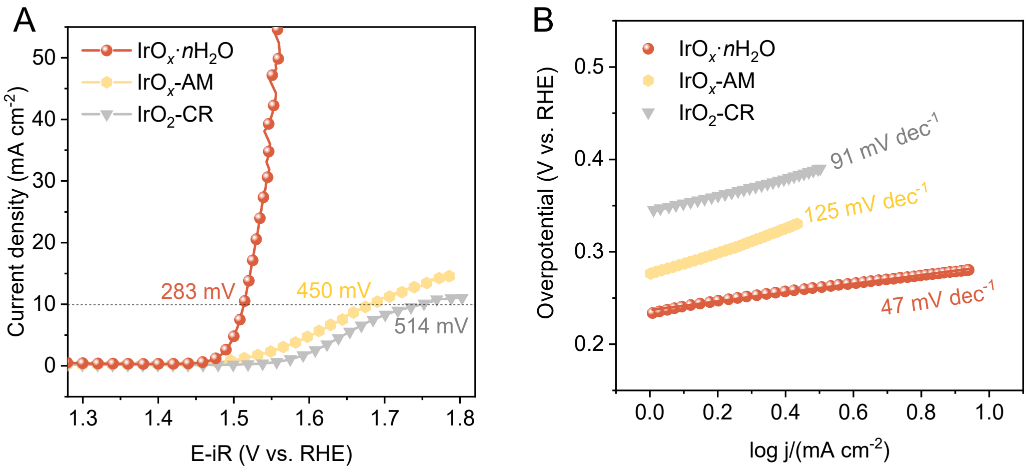

Fig. S11. Tafel plots analyses. (**A**) LSV curves normalized by geometric area, (**B**) Tafel plots from (**A**) of IrO$_x$·nH$_2$O, IrO$_x$-AM and IrO$_2$-CR.

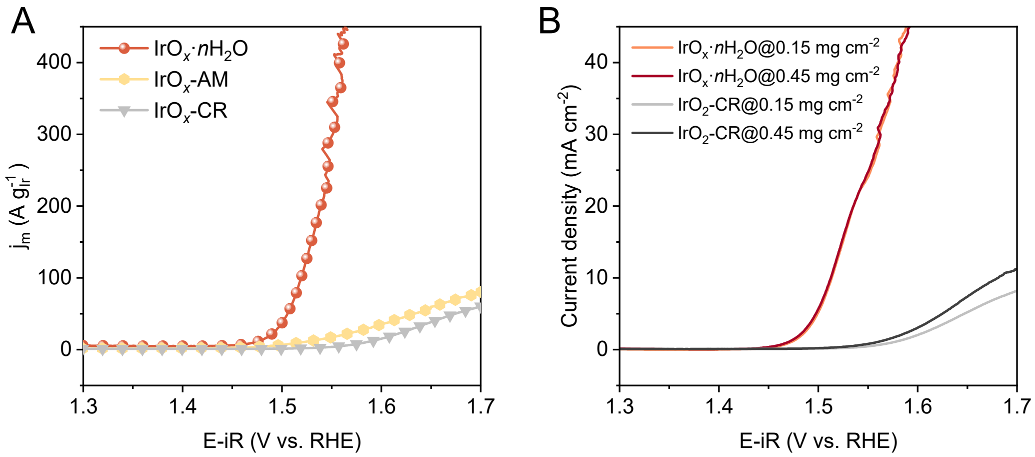

Fig. S12. Mass activity analyses. (**A**) Mass activity of IrO$_x$·nH$_2$O, IrO$_x$-AM and IrO$_2$-CR. (**B**) LSV curves normalized by geometric area with different loading**.**

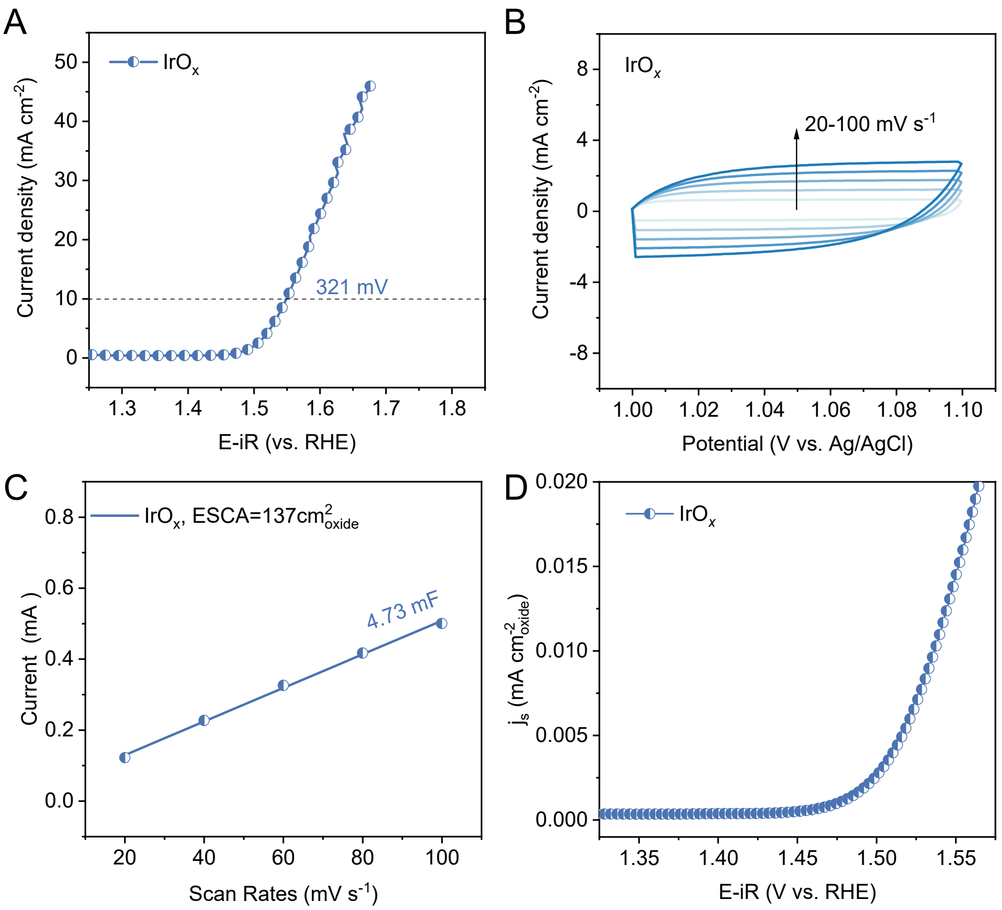

Fig. S13. Electrochemical measurements of IrO_x. (**A**) LSV curves normalized by geometric area. (**B**) CV curves with different scan rates in a non-Faradic region. (**C**) Double-layer capacitance (C_{dl}) represented by curve slope. (**D**) LSV curves normalized by ECSA.

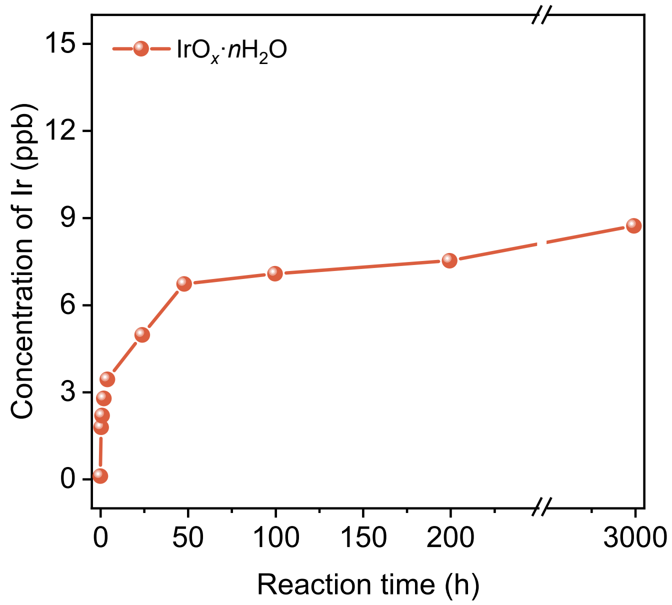

Fig. S14. Dissolved Ir of $IrO_x \cdot nH_2O$ in electrolyte following chronopotentiometry test (@10 mA cm^{-2}).

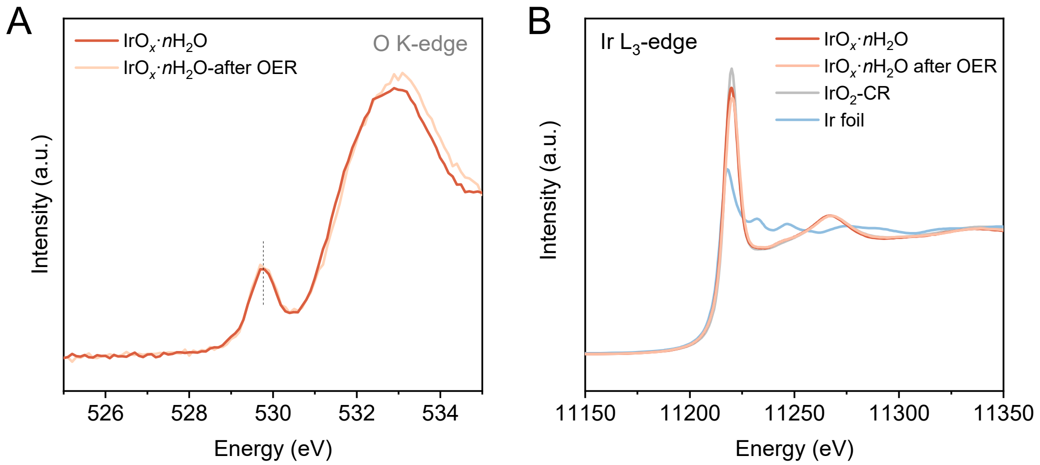

Fig. S15. Ex-situ XAS measurements on $IrO_x \cdot nH_2O$ prior to and following OER. (**A**) O K-edge and (**B**) Ir L_3-edge.

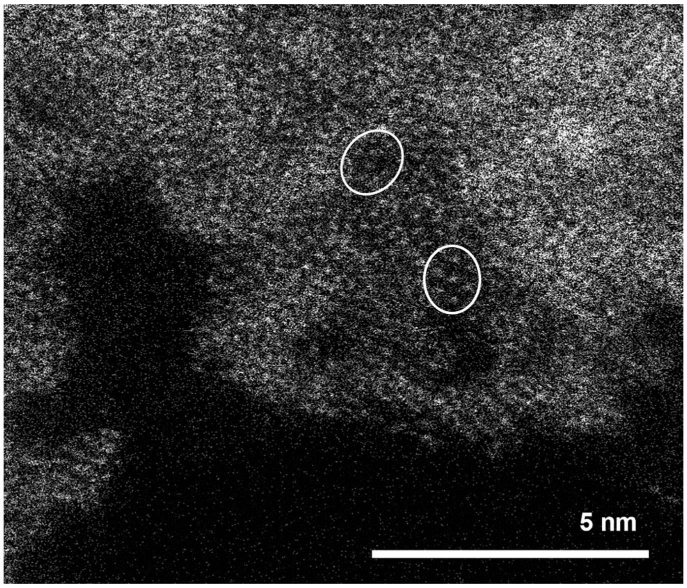

Fig. S16. HAADF-STEM image of $IrO_x \cdot nH_2O$ following OER test.

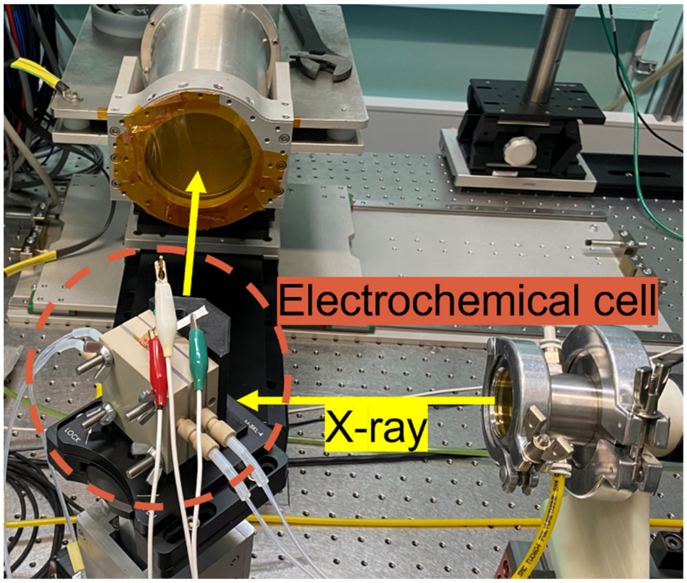

Fig. S17. Operando XAS setup.

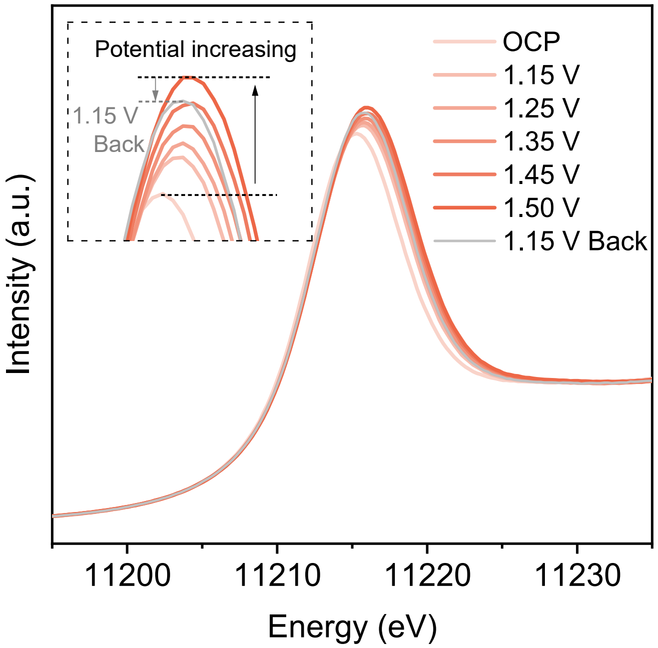

Fig. S18. Operando XANEs spectra of $IrO_x \cdot nH_2O$ under potential range of OCP ~ 1.50 V vs. RHE.

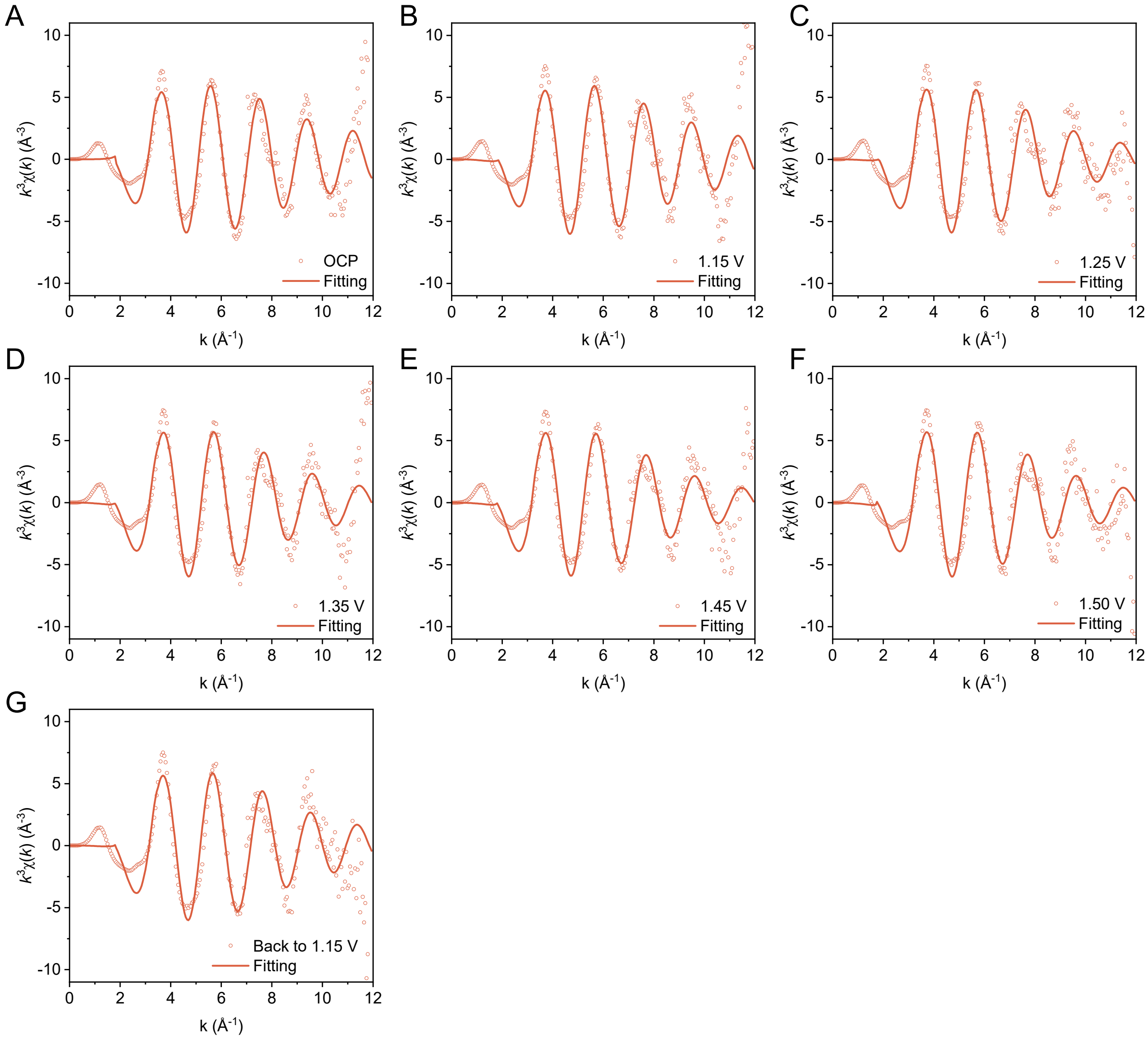

Fig. S19. Fitting data for operando XAS of $IrO_x \cdot nH_2O$. Ir L_3-edge EXAFS under different potentials fitting results at *R* space. Best-fit parameters are collected in Table S2.

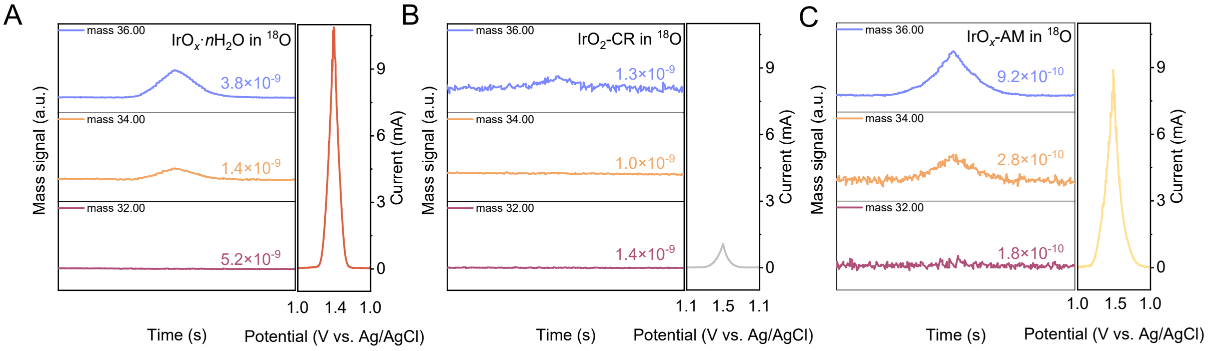

Fig. S20. DEMS results. Signals of O_2 products with corresponding CV curves in $H_2{}^{18}O$ supporting 0.05 M H_2SO_4 for (**A**) $IrO_x{\cdot}nH_2O$, (**B**) IrO_2-CR and (**C**) IrO_x-AM.

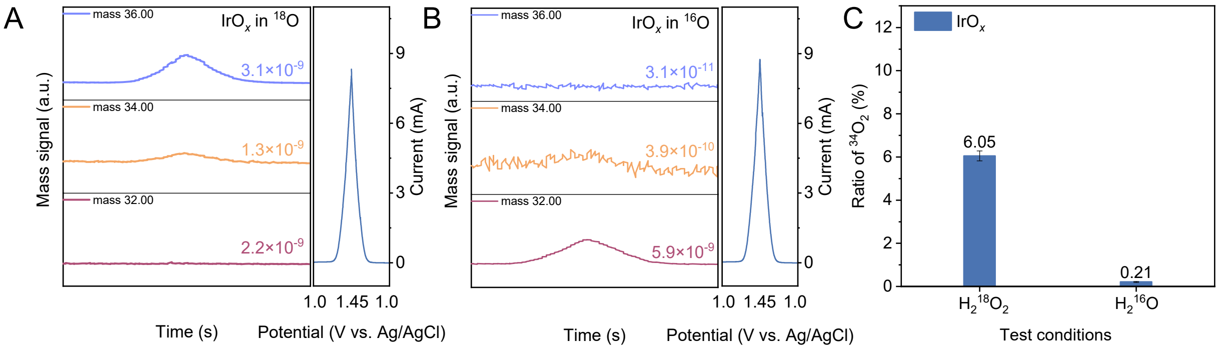

Fig. S21. Online DEMS signal for IrO_x under 0.05 M H_2SO_4 with different solvents. (**A**) $H_2{}^{18}O$ as solvent. (**B**) $H_2{}^{16}O$ as solvent. (**C**) Comparison of $^{34}O_2$ produced from 0.05 M H_2SO_4 using $H_2{}^{18}O$ and $H_2{}^{16}O$ as solvents.

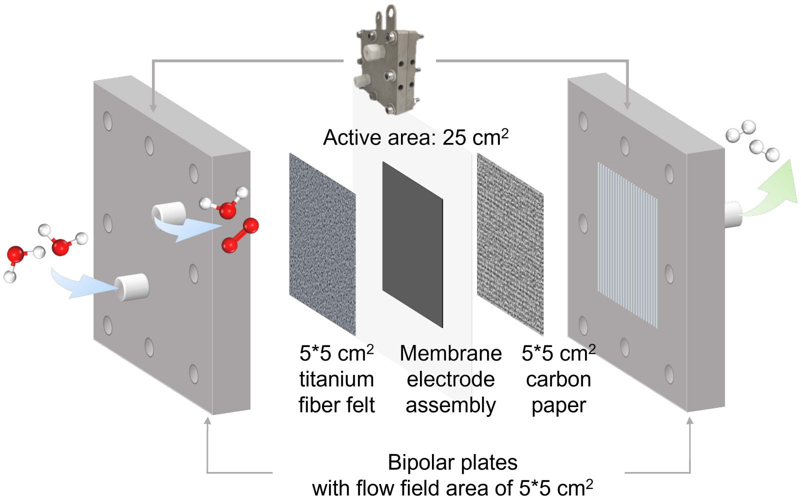

Fig. S22. Schematic for PEM electrolyzer.

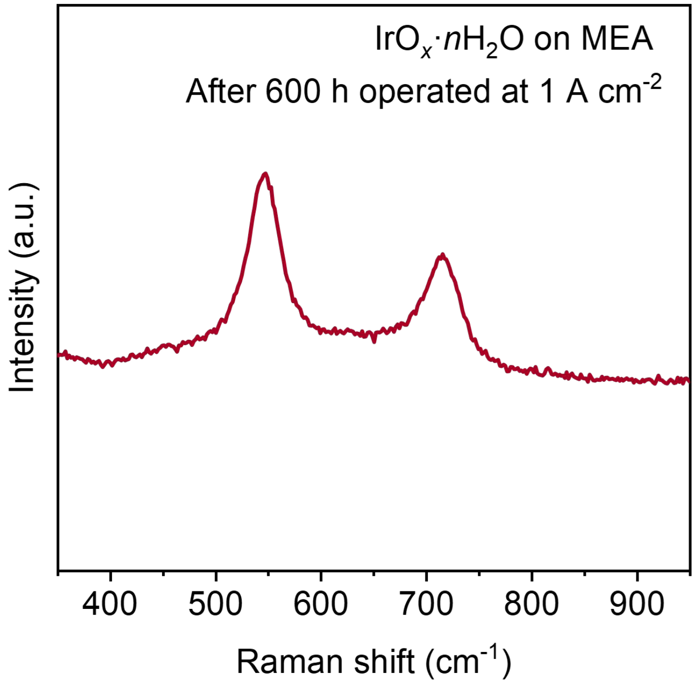

Fig. S23. Post Raman spectrum of MEA coated with $IrO_x \cdot nH_2O$.

Table S1. Comparative summary of $IrO_x \cdot nH_2O$ with selected Ir-based electrocatalysts for acidic OER reported in three-electrode system.

Catalyst	Electrolyte	Mass loading (mg cm^{-2})	Overpotential at corresponding j	Stability (@10 mA cm^{-2})	Reference
$IrO_x \cdot nH_2O$	0.1 M $HClO_4$	0.15	η_{10} = 283 mV	5700 h	This work
$IrO_x/SrIrO_3$	0.5 M H_2SO_4	--	η_{10} = 270-290 mV	30 h	(*30*)
Li−IrO_x	0.5 M H_2SO_4	0.125	η_{10} = 270 mV	10 h	(*34*)
IrO_2/GCN	0.5 M H_2SO_4	0.204	η_{10} = 276 mV	4 h (20 mA cm^{-2})	(*32*)
GB-$Ta_{0.1}Tm_{0.1}Ir_{0.8}O_{2-\delta}$	0.5 M H_2SO_4	0.04	η_{10} = 198 mV	500 h	(*37*)
Ir/Nb_2O_{5-x}	0.5 M H_2SO_4	0.128	η_{10} = 245 mV	105 h	(*38*)
$SrCo_{0.9}Ir_{0.1}O_{3-\delta}$	0.1 M $HClO_4$	0.255	$\eta_{16.3}$ = 320 mV	1.5 h	(*33*)
1T-IrO_2	0.5 M H_2SO_4	0.207	η_{10} = 197 mV	45 h (50 mA cm^{-2})	(*35*)
Ba_2YIrO_6	0.1 M $HClO_4$	0.015	η_{10} = 315 mV	1 h	(*29*)
Ir NSs	0.1 M $HClO_4$	0.204	η_{10} = 255 mV	8 h	(*36*)
6H-$SrIrO_3$	0.5 M H_2SO_4	0.90	η_{10} = 248 mV	30 h	(*31*)

Table S2. Operando EXAFS fitted parameters at Ir L_3-edge of $IrO_x \cdot nH_2O$. Artimise software together with standard procedure(s) was used for fits, and all $|\Delta E_0|$ were confirmed below 10 eV. *N* is the coordination number, σ^2 Debye-Waller factor, *R* coordination distance and *R*-factor relative error for fit.

E **(V vs. RHE)**	**Scattering path**	***N***	**Δ*R***	***R*** **(Å)**	σ^2 **(10^{-3} Å^2)**	***R*-factor**
OCP	Ir-O	5.667	0.069	2.027	5.5	0.0162
1.15	Ir-O	5.680	0.053	2.011	6.1	0.0195
1.25	Ir-O	6.056	0.046	2.004	7.7	0.0202
1.35	Ir-O	6.082	0.030	1.988	7.6	0.0161
1.45	Ir-O	6.139	0.028	1.986	8.1	0.0172
1.50	Ir-O	6.235	0.025	1.983	8.1	0.0206
1.15	Ir-O	5.949	0.045	2.003	6.8	0.0172

Table S3. Summary comparison of PEM electrolyzer performance with selected reports.

Anode catalyst	Mass loading /(mg cm^{-2})	Membrane	Operating temperature /°C	Cell voltage at 1 A cm^{-2}/V	Stability /(h @ A cm^{-2})	Cost /(USD per kg of H_2)	Energy consumption /kWh m^{-3} H_2	Reference
$IrO_x \cdot nH_2O$	2.0	N115	60	1.77	600@1.0	0.95	4.27	This work
IrO_2	3.0	N115	60	1.83	--	--	--	This work
Pt/ IrO_2	1.8	N117	60	1.81	--	--	--	(*47*)
GB-$Ta_{0.1}Tm_{0.1}Ir_{0.8}O_{2-\delta}$	0.2	N117	50	1.766	500@1.5	1.002	4.51	(*37*)
GB-$IrO_{2-\delta}$	0.2	N117	50	1.946	--	--	--	(*37*)
Ir/Nb_2O_{5-x}	1.80	N115	80	~1.65	2,000@2.0	0.984	4.43	(*38*)
Ir Black	2.0	N117	90	1.71	--	--	--	(*48*)
Ir AC/NN	1.0	N212	80	1.58	90@3	--	--	(*49*)
IrO_2 NN	1.0	N212	80	1.64	90@3	--	--	(*49*)
IrO_2 (Adams)	1.0	N212	80	1.66	< 90@3	--	--	(*49*)
IrO_x	1.5	N115	60	~1.70	--	--	--	(*50*)
$Ir_{0.2}Ru_{0.8}O_2$	1.5	N115	80	1.676	140@0.5	--	--	(*51*)
